# Transcatheter patent foramen ovale closure achieves high efficacy in migraine patients: a single-center retrospective cohort study in China

**DOI:** 10.3389/fneur.2026.1827976

**Published:** 2026-06-15

**Authors:** Hongzhan Cui, Jiao Wang, Tengyue Zhao, Hongjuan Liao, Jieqiong Zhang, Luyu Li, Ce Feng, Hongyu Xiao, Jialu Li, Ziying Chen

**Affiliations:** 1 Department of Cardiac Surgery, Second Hospital of Hebei Medical University, Shijiazhuang, China; 2 Hebei Normal University, Shijiazhuang, China; 3 Hebei University of Chinese Medicine, Shijiazhuang, China

**Keywords:** efficacy analysis, migraine, patent foramen ovale, percutaneous closure, real-world evidence, retrospective cohort study

## Abstract

**Introduction:**

Patent foramen ovale (PFO) is closely associated with migraine, and transcatheter PFO closure is a potential therapeutic intervention, yet large-scale real-world evidence in the Chinese population remains scarce.

**Methods:**

This single-center retrospective cohort study evaluated the efficacy, safety, and predictive factors of transcatheter PFO closure for migraine in Chinese patients, and explored the impact of procedural expertise evolution on treatment outcomes. A total of 974 consecutive migraine patients (15–65 years) who underwent successful transcatheter PFO closure at the Second Hospital of Hebei Medical University from July 2020 to December 2024 were enrolled, all with moderate-to-large right-to-left shunt (RLS) and a baseline Headache Impact Test-6 (HIT-6) score ≥60. All patients received standardized dual antiplatelet therapy (DAPT) post-procedure, and a cross-sectional follow-up was conducted between May 2024 and December 2024 (median follow-up: 16.0 months). Treatment response was defined as a ≥ 50% reduction in baseline HIT-6 score or monthly migraine days.

**Results:**

The overall migraine responder rate was 83.98%, with 59.90% achieving complete/near-complete relief (Grade I). Younger age was a strong predictor of better outcomes, with the highest responder rate in the 15–24 years group (89.67%) compared to the 45–54 years group (80.66%, *p* = 0.022). More recent procedure year was an independent predictor of higher success (OR = 0.89 per half-year delay, 95% CI: 0.81–0.97, *p* = 0.028), reflecting a significant learning curve effect. The procedure had a 100% technical success rate, with 3.49% minor complications and no major adverse events. Therapeutic benefit was sustained after DAPT discontinuation (responder rate 81.93% at >12 months). No significant differences were observed by sex, BMI, or occluder type.

**Discussion:**

In conclusion, transcatheter PFO closure is highly effective and safe for Chinese migraine patients with concomitant PFO, with sustained effects after DAPT withdrawal. Younger age and more recent procedural performance are key predictors of favorable outcomes.

## Highlights

- High overall migraine responder rate (83.98%) with 59.9% complete/near-complete relief after PFO closure in a Chinese migraine.
- Younger age (15–24 years) strongly predicts better outcomes (89.67%) compared to patients aged 45–54 years (80.66%) (*p* = 0.022).
- More recent procedure year independently predicts higher success (OR = 0.89 per half-year delay, 95% CI: 0.81–0.97, *p* = 0.028).
- Procedure is safe: 100% technical success, 3.49% minor complications, no major adverse events.
- Sustained benefit after DAPT discontinuation: responder rate 81.93% at >12 months.

## 1 Introduction

Migraine is a prevalent and disabling neurological disorder, characterized by recurrent attacks of moderate to severe headache and associated symptoms, which imposes a significant burden on quality of life and healthcare systems globally (1, 2). A substantial body of evidence has established an epidemiological link between migraine, particularly migraine with aura (MA), and PFO, with a prevalence of PFO as high as 40–60% in MA patients compared to 25% in the general population (3, 4). This strong association has spurred investigation into a potential causal relationship. Pathophysiological hypotheses proposed to explain this link include the paradoxical embolism of microthrombi, the shunting of vasoactive substances (e.g., serotonin) bypassing pulmonary filtration, and hemodynamic alterations with transient hypoxemia (5–7). These mechanisms provide a rationale for percutaneous PFO closure as a potential disease-modifying treatment aimed at the anatomical source of RLS.

While several randomized controlled trials (RCTs) [e.g., PRIMA trial (8), MIST trial (9), and PREMIUM trial (10)] have yielded neutral results for their pre-specified primary endpoints, these trials had significant methodological limitations that likely underestimated the true treatment effect. Early RCTs enrolled heterogeneous populations with varying PFO anatomy and migraine severity, failing to rigorously target patients with confirmed “PFO-driven” migraine; additionally, overly stringent primary endpoints, strong placebo effects, and relatively short follow-up durations likely attenuated the observed efficacy signal, even as secondary analyses consistently showed benefits in key subgroups. In contrast, pooled analyses and real-world observational studies consistently suggest significant therapeutic benefit in highly selected patients (11, 12).

The importance of patient selection is further highlighted by a cross-sectional study by Rundek et al. (13), which found no significant association between PFO and migraine in a community-based cohort, indicating unselected populations may not benefit from closure. A key challenge is identifying which patients are most likely to benefit. Current evidence suggests that younger age, presence of aura, and larger shunt size predict better outcomes, but consistent predictive models are lacking (14). Notably, patients with high-risk anatomical features (e.g., large shunt, atrial septal aneurysm) appear to derive superior results (15, 16).

Unlike these earlier studies that enrolled broader populations, our study focused on strictly defined patients with severe migraine (HIT-6 ≥ 60) and moderate-to-large RLS (cTCD grade ≥II), a high-risk subgroup more likely to derive procedural benefit. Nonetheless, large-scale, long-term real-world data from Chinese populations remain scarce for confirming its generalizable effectiveness and safety profile in diverse clinical practice settings. The clinical impact of evolving procedural expertise, a factor rarely quantified in previous studies, also remains unclear.

To address these evidence gaps, this large single-center retrospective cohort study aimed to assess the real-world efficacy and safety of transcatheter PFO closure for migraine in a Chinese population, thereby providing robust real-world evidence for this population. A primary objective was to investigate whether readily available clinical factors, specifically patient age and the evolution of procedural expertise (proxied by the chronological sequence of surgery), are independently associated with treatment response, with the goal of contributing to more refined patient selection criteria.

## 2 Methods

### 2.1 Study design and patients

This was a single-center, retrospective cohort study. The analysis included 974 consecutive patients aged 15–65 years who underwent successful transcatheter PFO closure at the Second Hospital of Hebei Medical University between July 2020 and December 2024. All patients presented with migraine as the primary symptom. The clinical diagnosis of migraine was established by neurologists according to the International Classification of Headache Disorders, 3rd edition (ICHD-3) criteria and was required to have a history of more than one year (17). Strict inclusion criteria were applied: a baseline HIT-6 score ≥60 and moderate-to-large right-to-left shunt (cTCD grade ≥II). This was supplemented by a headache diary documenting monthly migraine days, attack frequency, duration, and severity (Visual Analogue Scale, VAS). The presence of a RLS at the atrial level was confirmed by contrast-enhanced transcranial Doppler (cTCD) and/or contrast transthoracic echocardiography (cTTE) (18). A cTCD grade ≥ II (i.e., >20 microbubble signals within 10 s of injection) was defined as a clinically significant moderate-to-large RLS shunt (19). Key exclusion criteria were: (1) other identifiable intracranial causes of headache (e.g., aneurysm, arteriovenous malformation on CT/MRI); (2) significant concomitant cardiac disease (e.g., atrial septal defect, valvular disease, pulmonary arteriovenous fistula); (3) contraindications to the procedure or antiplatelet therapy; (4) coagulation disorders or chronic anticoagulation; (5) severe renal/hepatic dysfunction, malignancy, or psychiatric disorders; (6) cognitive, hearing, or vision impairment hindering communication; and (7) incomplete follow-up data or poor compliance. This study was conducted in accordance with the Declaration of Helsinki and approved by the Research Ethics Committee of Fuwai Hospital, Chinese Academy of Medical Sciences (approval No. 2024-C042). Written informed consent for clinical treatment was obtained from all patients before the procedure. Since this consent did not cover the use of clinical data for retrospective research, the ethics committee waived the requirement for additional informed consent.

### 2.2 Preprocedural evaluation, procedure, and follow-up

Prior to closure, all patients underwent a comprehensive evaluation. The presence and functional significance of RLS were primarily established by cTCD and cTTE, as detailed in the inclusion criteria. Transesophageal echocardiography (TEE) was selectively employed to obtain precise anatomical definition of the PFO (e.g., resting diameter, tunnel length) and to assess for associated high-risk features such as an atrial septal aneurysm (ASA) or a prominent Eustachian valve, particularly in cases with complex anatomy or when non-invasive imaging was inconclusive. While TEE offers superior anatomical detail, its use was individualized based on clinical judgment and patient tolerance. This multimodality imaging approach, centered on functional shunt detection (cTCD/cTTE) supplemented by selective anatomical clarification (TEE), was used for definitively confirming the cardiac origin of the RLS and excluding other sources.

The PFO closure procedure was performed per standard clinical practice. Device selection (including Amplatzer PFO Occluder from Abbott Co. and domestic nitinol occluder models from Beijing Huayi Shengjie Co.) was individualized based on anatomical measurements from TEE/cTTE and operator judgment. The procedure involved femoral venous access, crossing the PFO under fluoroscopic and echocardiographic guidance, device deployment, and confirmation of stability via standardized “push-pull” and “jiggle” tests before release. All procedures were performed by an experienced team, and procedural success was defined as successful device implantation without major complications. All patients received dual antiplatelet therapy (DAPT; aspirin 100 mg/day and clopidogrel 75 mg/day) post-procedure, with clopidogrel discontinued at 6 months and aspirin continued for 1 year. This DAPT regimen was administered in accordance with the Chinese Expert Consensus on Patent Foramen Ovale Closure (2023) to reduce the risk of periprocedural thrombosis and potentially contribute to migraine symptom relief.

A systematic follow-up protocol was implemented using a cross-sectional design. All patients underwent a single follow-up assessment between May 2024 and December 2024. Consequently, the postoperative follow-up duration varied according to the patient’s surgery date: patients who underwent surgery in later years (e.g., 2024) had shorter follow-up durations, while those who underwent surgery in earlier years (e.g., 2020–2022) had longer follow-up durations, with some extending up to 48 months. Because the exact follow-up date was not recorded due to the retrospective nature of the study and routine clinical follow-up practice, patients were grouped into eight sequential half-year batches based on their surgery date (Batch 1: 2024.07-2024.12; Batch 2: 2024.01-2024.06; Batch 3: 2023.07-2023.12; Batch 4: 2023.01-2023.06; Batch 5: 2022.07-2022.12; Batch 6: 2021.07-2021.12; Batch 7: 2021.01-2021.06; Batch 8: 2020.07-2020.12). These batches serve as a proxy for the range of postoperative follow-up intervals in subsequent analyses. At each follow-up visit, patients completed the HIT-6 scale and provided information on migraine status via structured interviews with standardized questionnaires. For patients assessed at later time points, the timing of initial symptom relief (e.g., immediate, within 1 month, within 3 months) was collected retrospectively. Based on the surgery date and the uniform follow-up window (May–December 2024), patients were further classified into two groups for subanalysis: those assessed within 12 months post-surgery (active DAPT period) and those assessed after 12 months (post-DAPT period). For the purpose of reporting summary follow-up statistics, the postoperative follow-up duration for each patient was estimated by assuming a common cutoff date of December 31, 2024, calculated as the interval from the surgery date to this cutoff date, and is expressed in months.

### 2.3 Outcomes and definitions

The co-primary efficacy measures were the change in the HIT-6 score and the change in monthly migraine days from baseline. These measures were assessed at the single follow-up visit for each patient, comparing their current status with retrospectively collected baseline data. Treatment response (effective improvement) was defined as meeting either of the following criteria at the time of follow-up assessment: *a* ≥ 50% reduction from the baseline HIT-6 score or *a* ≥ 50% reduction in monthly migraine days compared to baseline. Patients not meeting either criterion at follow-up were classified as having “no significant improvement”, a definition pre-specified for this study to ensure consistent outcome assessment. Secondary endpoints included: (1) time to symptomatic relief (categorized as immediate, within 1 month, within 3 months, within 6 months, or >6 months); assessed retrospectively at the follow-up visit; (2) grade of improvement based on the HIT-6 score at the time of follow-up assessment: Grade I (complete or near-complete relief), score 36–45; Grade II (substantial relief), score 46–60; Grade III (mild relief), score >60; and (3) the presence and grade of residual shunt on follow-up echocardiography at the time of each patient’s follow-up visit.

Safety was assessed by recording all periprocedural and follow-up complications. Major complications were defined as device embolization, cardiac tamponade, stroke, transient ischemic attack (TIA), or need for surgical intervention. Minor complications included puncture site hematoma (grade I/II per clinical grading), transient fever (37.5 °C–38.0 °C), and benign, self-limiting arrhythmias (atrial/ventricular premature beats), with all complications documented and graded per standardized clinical criteria.

### 2.4 Statistical analysis

Continuous variables are presented as mean ± standard deviation (SD) or median (interquartile range, IQR) after normality testing (Shapiro–Wilk test): if data were normally distributed, mean ± SD was used; otherwise, IQR was used. Continuous variables were compared using paired *t*-tests (for normally distributed data) or Wilcoxon signed-rank tests (for non-normally distributed data) for each follow-up cohort separately, where follow-up cohorts were grouped by the half-year batches of surgery dates. Comparisons across different follow-up time points were performed using one-way ANOVA or Kruskal–Wallis test as appropriate, with Bonferroni correction was applied for multiple post-hoc comparisons. Categorical variables are presented as frequencies (percentages) and were compared using the Chi-square test or Fisher’s exact test. Pre- and post-procedural HIT-6 scores were compared separately for each follow-up cohort. Univariate analyses (Chi-square, Mann–Whitney *U* test) were performed to evaluate associations between clinical variables (age, sex, BMI, procedure time period, occluder type) and the binary treatment response. Variables with *p* < 0.1 in univariate analysis were entered into a multivariable logistic regression model (forward: LR method) to identify independent predictors of treatment response. The model’s discriminative ability was assessed using the area under the receiver operating characteristic (ROC) curve, and goodness-of-fit was evaluated with the Hosmer–Lemeshow test. A separate linear regression model was used to analyze the correlation between the continuous responder rate and the procedure time period (grouped in 6-month batches). A two-sided *p*-value <0.05 was considered statistically significant. All analyses were performed using SPSS version 29.0 (IBM Corp., Armonk, NY, USA) with detailed operation steps recorded for reproducibility.

To assess the robustness of the primary findings, we performed several sensitivity analyses. First, to disentangle the procedural effect from the potential confounding effect of antiplatelet therapy on treatment efficacy, we stratified patients based on their estimated follow-up duration relative to DAPT completion (≤12 months *vs.* >12 months) and re-examined the association between procedure time batch and migraine improvement within each stratum. Second, we repeated the multivariable logistic regression model excluding the smallest batch (Batch 8, *n* = 5) to ensure that the observed learning curve effect was not driven by this outlier. The results of these sensitivity analyses were consistent with those of the main analysis, supporting the robustness of our conclusions. Detailed results of the DAPT-stratified analysis are presented in Section 3.2.

## 3 Results

### 3.1 Patient characteristics and overall efficacy

A total of 1,036 consecutive patients with migraine and PFO were screened for eligibility. Of these, 62 were excluded: 45 did not meet the inclusion criteria (e.g., HIT-6 score <60, absence of significant RLS), 12 had incomplete follow-up data, and 5 declined to participate. The remaining 974 patients were included in the final analysis. Baseline demographic, clinical, and procedural characteristics are summarized in Table 1. The study population had a mean age of 38.6 ± 13.6 years, with the largest proportion (28.5%) aged 36–45 years. A total of 641 patients (65.8%) were female, resulting in a female-to-male ratio of approximately 2:1. Mean BMI was 24.4 ± 4.1 kg/m^2^. The mean preoperative HIT-6 score was 65.2 ± 6.1, reflecting a severe impact of headache on daily life; younger age groups tended to have higher baseline HIT-6 scores. All patients had moderate-to-large RLS (Grade II or III) as confirmed by preprocedural imaging—a key inclusion criterion. Atrial septal aneurysm was present in 276 patients (28.3%). Occluder devices were primarily Nitinol-based, with domestic devices used in 56.9% of cases and imported devices in 43.0%; two patients (0.2%) received a bioabsorbable device. Device model selection was individualized based on anatomical assessment. There were no missing data for any of the variables analyzed in this study.

**Table 1:** Baseline characteristics of the study population (*N* = 974).

Characteristics	Value
Age, yrs	38.6 ± 13.5
Female sex, *n* (%)	641 (65.8%)
Body mass index, kg/m^2^	24.4 ± 4.1
Atrial septal aneurysm, *n* (%)	276 (28.3%)
Occluder type, *n* (%)	974(100%)
Domestic (Nitinol)	554 (56.9%)
Imported (Nitinol)	418 (42.9%)
Bioabsorbable	2 (0.2%)
Pre-procedural HIT-6 Score	65.2 ± 6.1

Patients were grouped into eight sequential half-year batches according to their surgery date (Batch 1: 2024.07-2024.12, *n* = 153; Batch 2: 2024.01-2024.06, *n* = 251; Batch 3: 2023.07-2023.12, *n* = 183; Batch 4: 2023.01-2023.06, *n* = 226; Batch 5: 2022.07-2022.12, *n* = 92; Batch 6: 2021.07-2021.12, *n* = 33; Batch 7: 2021.01-2021.06, *n* = 31; Batch 8: 2020.07-2020.12, *n* = 5). Due to the cross-sectional follow-up design (all patients were assessed once between May 2024 and December 2024), the actual postoperative follow-up duration varied according to surgery date, ranging from a few months to up to 48 months across the cohort. Based on a uniform cutoff date of December 31, 2024, the estimated median follow-up duration was 16.0 months (IQR: 8.5–20.9 months). This distribution allowed us to capture short-term, mid-term, and long-term outcomes.

**Figure 1:**
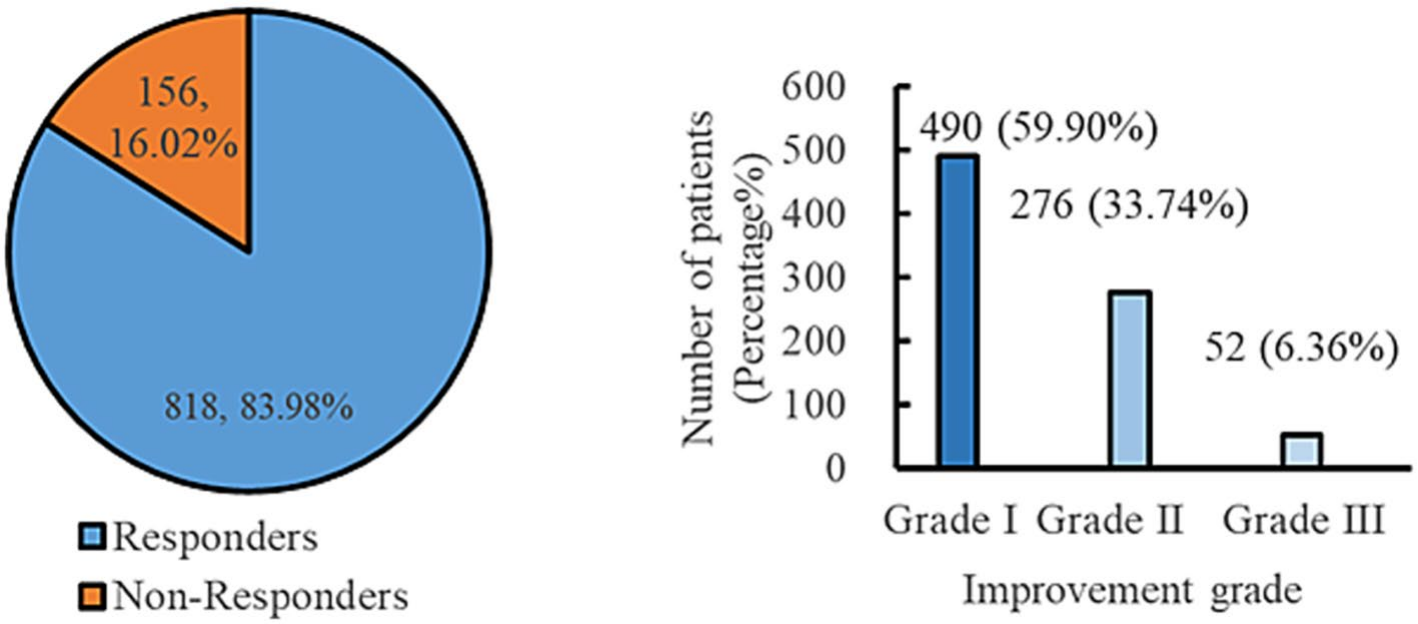
Overall rates of migraine improvement and distribution of response grades after PFO closure (*N* = 974). **(A)** Proportion of patients achieving the composite endpoint of migraine improvement (≥50% reduction in HIT-6 score or monthly migraine days). **(B)** Distribution of improvement grades among responders (*n* = 818), based on postoperative HIT-6 score ranges.

At follow-up, 818 patients (83.98%) met the criteria for migraine improvement, while 156 patients (16.02%) showed no significant response. This overall response rate is consistent with rates reported in other large cohorts, such as the 80% improvement noted by Rigatelli et al. (20), and the slightly higher rate observed here may be attributable to differences in population selection and follow-up management in our study. Among these responders, migraine improvement was further stratified by degree according to HIT-6 score reduction: 490 patients (59.90% of responders, 50.3% of the total cohort) achieved Grade I improvement (near-complete or complete resolution), 276 patients (33.74%) attained Grade II improvement (substantial but incomplete relief), and only 52 patients (6.36%) were classified as Grade III improvement (mild relief), as shown in Figure 1. This distribution indicates that the majority of responders experienced marked clinical benefit, with nearly 60% achieving a level of relief consistent with functional recovery.

**Figure 2:**
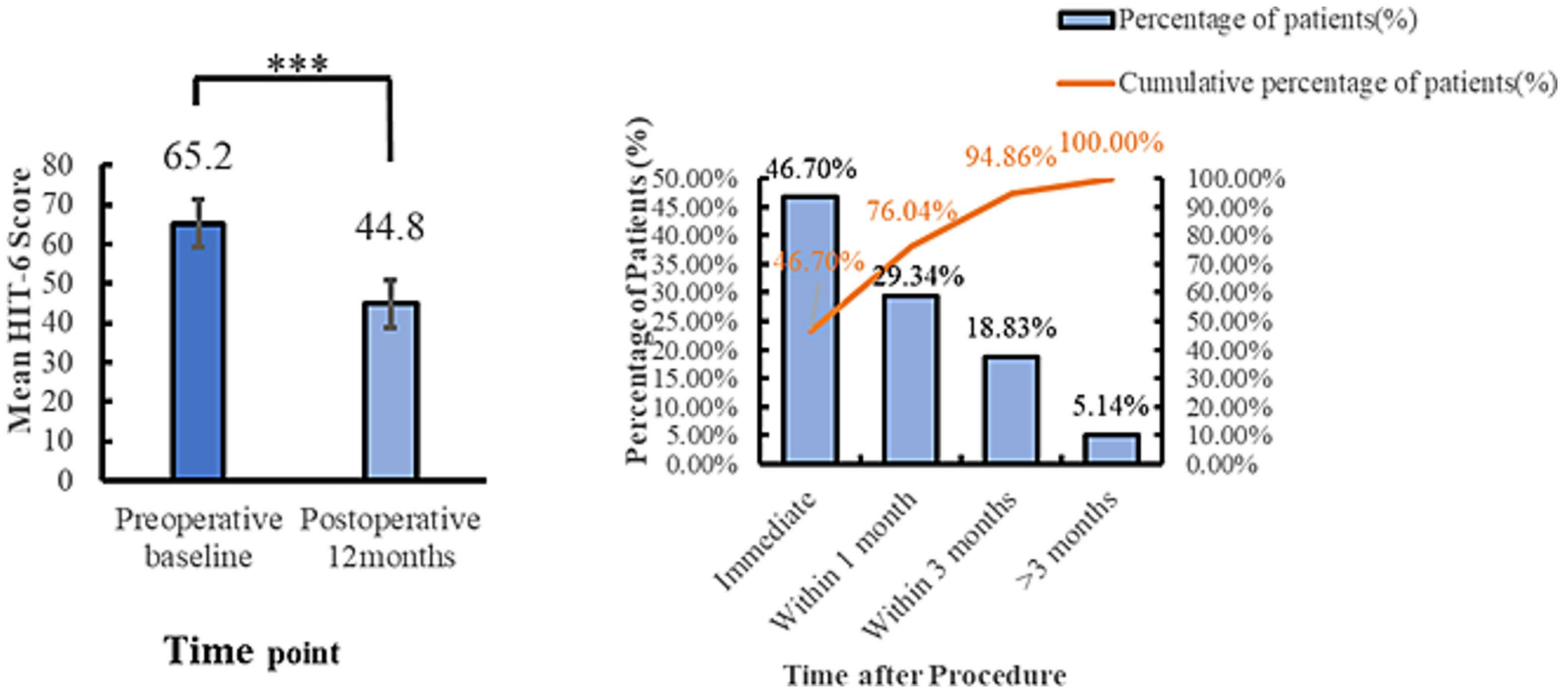
Magnitude and time course of symptom relief after PFO closure. **(A)** Comparison of preoperative and postoperative HIT-6 scores among patients assessed at the 12-month follow-up (mean ± SD). ***p < 0.001 *vs.* preoperative. **(B)** Distribution of patient-reported time to initial symptom relief, assessed retrospectively at the follow-up visit.

Supporting this categorical assessment, among patients assessed at the 12-month follow-up, the mean HIT-6 score demonstrated a significant decrease from 65.2 ± 6.1 preoperatively to 44.8 ± 6.0 (mean reduction: 20.4 points; *p* < 0.001), as shown in Figure 2A. Concurrently, objective migraine burden metrics also improved substantially. The monthly migraine days were significantly reduced compared to baseline among both the 12-month and 24-month follow-up cohorts (*p* < 0.05 for both). A total of 78.2% of patients across all follow-up cohorts met the effective response criteria (≥50% reduction in monthly migraine days or attack frequency) at their respective follow-up assessments, including a subgroup who achieved complete migraine cessation. This therapeutic benefit was consistently observed across follow-up cohorts.

The time course of symptom relief was assessed retrospectively. Within the responder group, 76.04% (622/818) reported improvement within the first postoperative month, with 46.70% reporting immediate postoperative relief (Figure 2B). Delayed improvement beyond 3 months was reported by only 5.14% of responders. While these retrospective data suggest that the primary therapeutic effect occurs early after shunt elimination, they should be interpreted with caution due to potential recall bias.

### 3.2 Factors associated with treatment response

**Figure 3:**
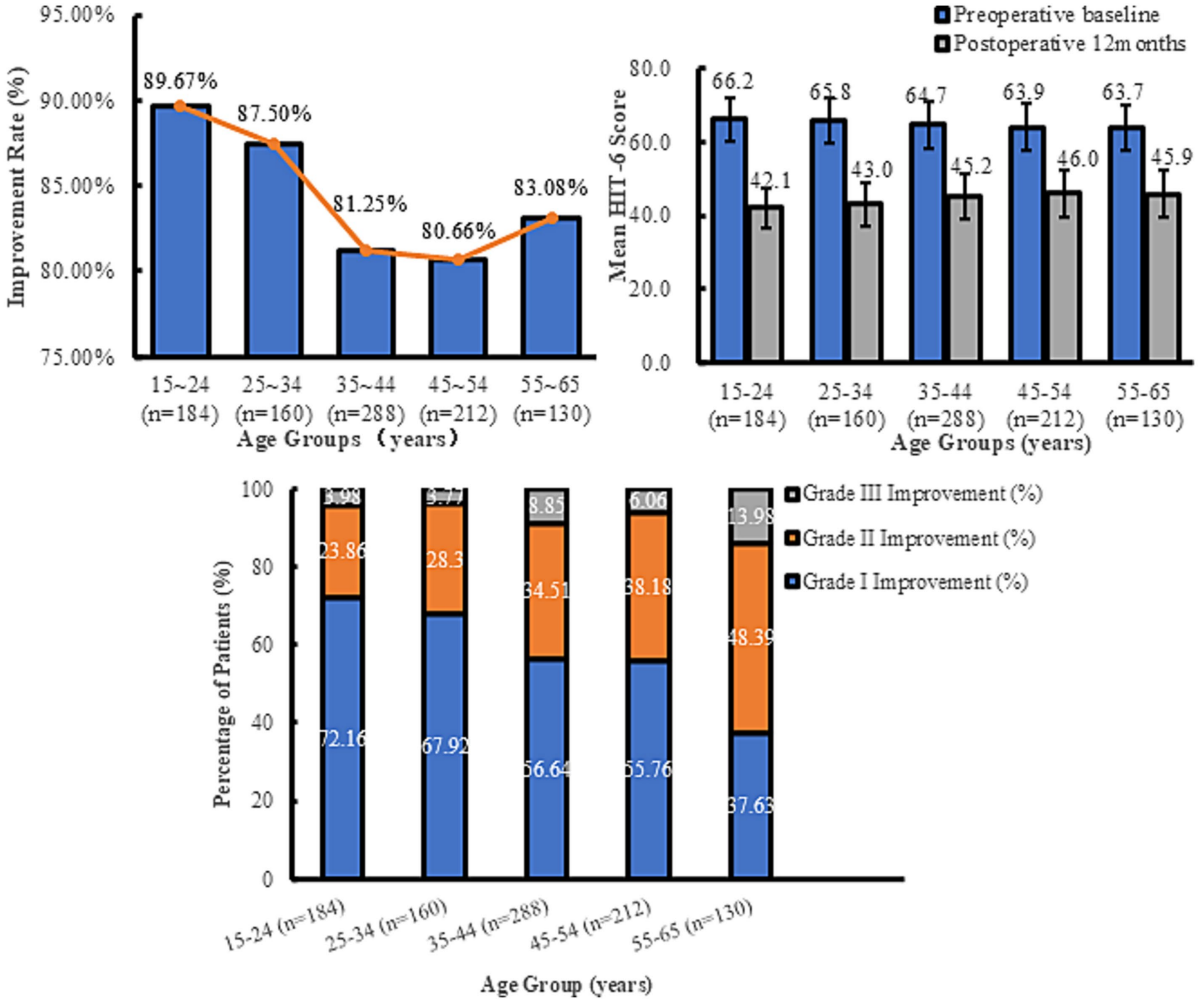
Association between age and clinical efficacy. **(A)** Migraine responder rate by age group after PFO closure (*N* = 974). **(B)** HIT-6 score before and after closure among patients assessed at 12-month follow-up. **(C)** Distribution of improvement grades by age group.

Analysis of clinical variables revealed several associations with treatment response. A significant inverse relationship was observed between patient age and the likelihood of achieving a treatment response (*p* = 0.022). As shown in Figure 3A, the responder rate was highest in the youngest cohort (aged 15–24 years) at 89.67% and demonstrated a gradual decline with increasing age to 87.50% (25–34 years), 81.25% (35–44 years), 80.66% (45–54 years), and 83.08% (55–65 years). Correspondingly, the magnitude of reduction in HIT-6 scores was most pronounced in younger patients, with a mean decrease of 24.1 points in the 15–24 year group, compared to 17.8 points in the 55-65 year group (Figure 3B). Furthermore, the quality of response differed by age (Figure 3C): the proportion of patients achieving Grade I (complete or near-complete) relief was 72.16% in the 15–24 year group but declined markedly to 37.63% in the 55–65 year group. While a negative correlation trend was confirmed by linear regression (*B* = −0.005), and age was a significant factor in univariate analysis, it was not retained as an independent predictor in the multivariable logistic regression model (OR = 0.95, *p* = 0.076), suggesting its effect may be mediated by or interact with other variables.

**Figure 4:**
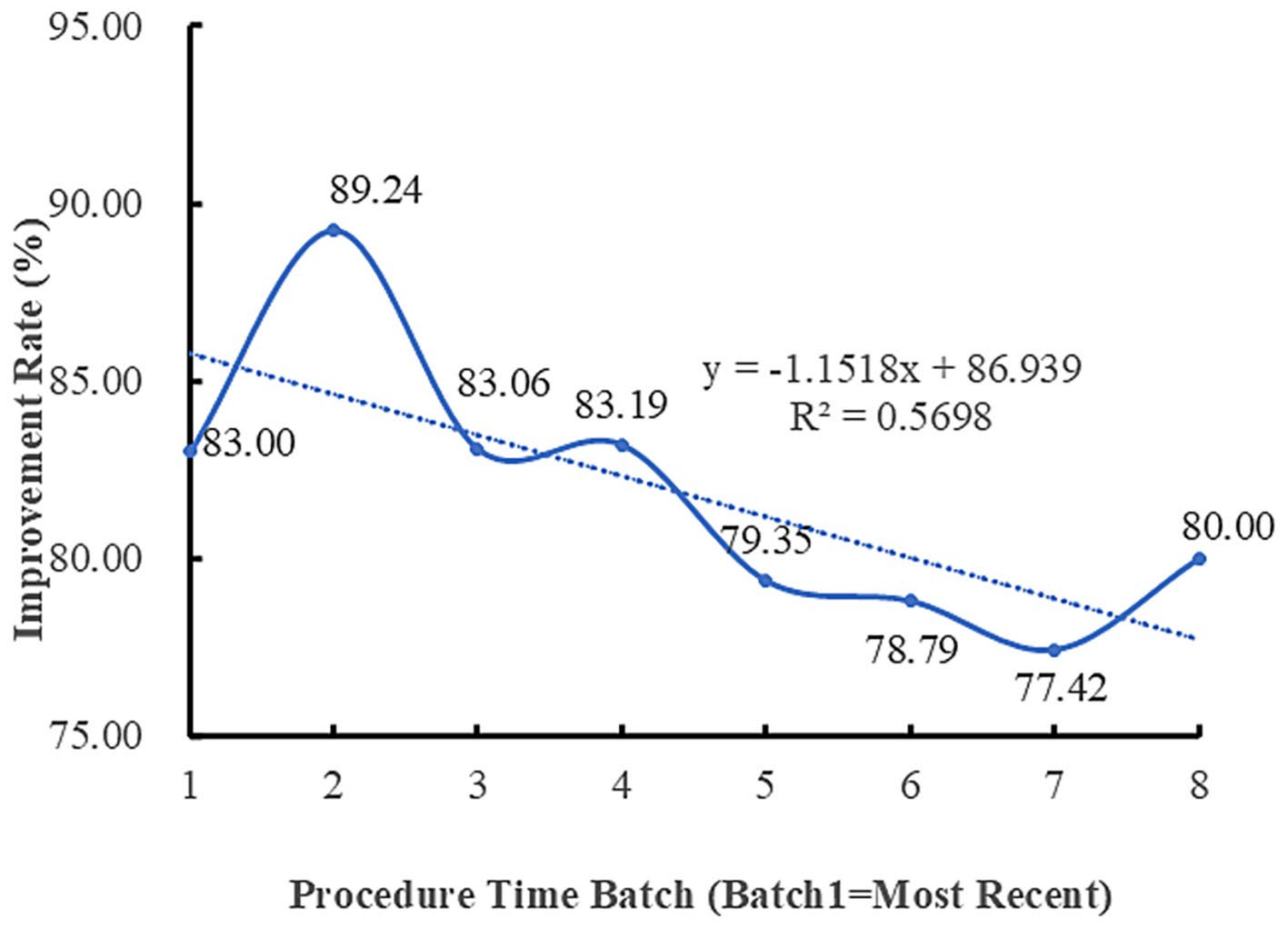
The impact of procedure time on efficacy. Scatter plot of procedure time batch *vs.* migraine responder rate, with regression line. Batches are numbered chronologically, with Batch 1 being the most recent (2024.07-2024.12) and Batch 8 the earliest (2020.07-2020.12). In detail, Batch 1: 2024.07-2024.12; Batch 2: 2024.01-2024.06; Batch 3: 2023.07-2023.12; Batch 4: 2023.01-2023.06; Batch 5: 2022.07-2022.12; Batch 6: 2021.07-2021.12; Batch 7: 2021.01-2021.06; Batch 8: 2020.07-2020.12.

When examining the effect of the chronological period of the procedure, a clear temporal trend emerged. Procedures were grouped by half-year intervals from 2020 to 2024. Batches were numbered chronologically, with Batch 1 representing the most recent period (2024.07–2024.12) and Batch 8 the earliest (2020.07-2020.12). As shown in Figure 4, the highest responder rates were observed in the most recent batches (Batch 1–3, ranging from 83.00% to 89.24%) and gradually declined in earlier batches, with the lowest rate observed in Batch 7 (77.42%); batch 8 had a responder rate of 80.00%, consistent with the data in Figure 4. All patients in this study received a standardized post-procedure DAPT regimen (aspirin 100 mg/d for 1 year and clopidogrel 75 mg/d for 6 months). The anti-inflammatory and analgesic properties of aspirin, combined with the antiplatelet effects of both agents potentially reducing microembolic load, may have contributed to early symptom relief. This pharmacological effect is particularly relevant for patients in recent batches, as their follow-up assessments were conducted within the first-year post-surgery (e.g., at 1, 3, or 6 months), when they were still on active DAPT. In contrast, patients in earlier batches were assessed at later time points (e.g., 12 or 24 months), after DAPT had been discontinued.

To exclude the pharmacological effect of DAPT on the efficacy of PFO closure and evaluate the durable effect of mechanical closure alone, we compared responder rates between patients assessed during the active DAPT period (follow-up ≤12 months) and those assessed after DAPT completion (follow-up >12 months). Among patients assessed after DAPT completion (n = 570), the responder rate remained substantial (81.93%), indicating that the therapeutic benefit of mechanical closure was sustained even after drug withdrawal. Beyond the drug effect, a significant inverse correlation was observed between procedure batch and responder rate among patients assessed after DAPT completion. Within the earlier batches (Batch 4–8), where most patients were assessed after DAPT completion, the responder rate demonstrated a consistent declining trend as the procedural date was earlier (from 83.19% to 77.42%), excluding Batch 8 (*n* = 5) due to its small sample size and volatile responder rate (80.00%), which could distort the trend. Linear regression confirmed this negative correlation between the procedure batch sequence (with later batches coded with higher numbers) and the responder rate, indicating that even after accounting for the uniform DAPT background and focusing on patients assessed after drug withdrawal, earlier procedure date was associated with a lower probability of sustained migraine improvement at follow-up (OR = 0.89 per half-year delay, 95% CI: 0.81–0.97, *p* = 0.028), meaning that the probability of treatment response increases by 11% for each half-year delay in the procedure batch. This quantifiable learning curve effect underscores that technical proficiency and systematized care are key drivers of success in PFO closure for migraine in the Chinese clinical setting, a novel finding for this population. These findings were robust in sensitivity analyses that stratified by DAPT period and excluded the smallest batch (see Statistical analysis).

In contrast, no statistically significant difference in responder rates was found between sexes (84.5% in females *vs.* 82.9% in males, *p* = 0.318) or between BMI groups dichotomized at 24 kg/m^2^ (*p* = 0.451). Although descriptive analysis indicated that patients with suboptimal outcomes had BMIs clustering in the 23.8–25.1 kg/m^2^ range (the upper normal to overweight threshold), this variable did not achieve significance in the univariate or multivariable models. This finding contrasts with some prior hypotheses linking obesity to poorer outcomes in PFO closure, suggesting that in this Chinese migraine-specific cohort, BMI may not be a primary determinant of procedural success. Similarly, the type or model of the occluder device used (including manufacturer and size) did not show a statistically significant effect on clinical efficacy in our analysis.

### 3.3 Safety outcomes

The procedure demonstrated a high safety profile in this large migraine patient cohort, which is crucial for establishing its viability as a routine therapeutic option in this population (21). The procedural technical success rate was 100%, with no periprocedural deaths or major complications, including cardiac tamponade, device embolization, or major vascular events, as summarized in Table 2. A total of 34 patients (3.49%) experienced minor complications during the hospitalization or within one-month post-procedure. These included puncture site hematoma (*n* = 21, 2.16%), transient arrhythmias (*n* = 8, 0.82%), and low-grade fever (*n* = 5, 0.51%); transient arrhythmias were primarily atrial or ventricular premature beats attributed to mechanical stimulation during catheter manipulation, and low-grade fever (37.5 °C–38.0 °C) was likely related to a mild inflammatory response to the device. All minor complications resolved spontaneously or with simple intervention (e.g., local compression for hematoma, observation for fever) without sequelae. Notably, during the follow-up period of up to 24 months, there were no occurrences of device-related thrombosis, new-onset atrial fibrillation, device displacement, recurrent stroke, or transient ischemic attack (TIA). Importantly, 16 patients (1.6%) reported new-onset or transient worsening of migraine symptoms within the first 3 months post-procedure, which subsequently resolved spontaneously in all cases. All of these patients were ultimately classified as responders based on the ≥50% reduction in HIT-6 score or monthly migraine days at their follow-up assessment, indicating that transient early worsening does not preclude long-term benefit. Analysis of risk factors showed no significant association between complication occurrence and patient age, sex, or occluder type (*p* > 0.05), confirming the consistent safety of PFO closure across different subgroups in the Chinese population. The absence of severe long-term complications supports the procedural safety profile of PFO closure in this migraine patient cohort.

**Table 2:** Procedural and follow-up safety outcomes (*n* = 974).

Category	Safety event	*n*	%	Management/outcome
Procedure success	Successful device implantation	974	100%	N/A
Major peri-procedural complications	Death, cardiac tamponade, device embolism, major bleeding	0	0%	None
Total minor complications	-	34	3.49%	All resolved without sequelae
Follow-up events	New-onset or worsened migraine within 3 months	~16	~1.6%	Gradually alleviated over time
Long-term follow-up (24 months)	Device migration, stroke, TIA, device-related thrombosis	0	0%	None

## 4 Discussion

The association between PFO and migraine, particularly with aura, is well-established, and transcatheter PFO closure has emerged as a potential intervention for refractory cases (22–24). However, several negative/neutral studies have challenged this benefit: Mattle et al. (8) reported neutral primary outcomes in an RCT of migraine with aura, which may be attributed to broad, unselected enrollment, and Rundek et al. (13) observed no PFO-migraine association in a general population. Despite these conflicting reports, real-world evidence increasingly supports the therapeutic role of PFO closure. This large real-world cohort study of 974 Chinese patients demonstrates that transcatheter PFO closure is highly effective and safe for migraine relief, with an overall responder rate of 83.98% and 59.90% achieving Grade I (complete/near-complete) relief. These outcomes align with or exceed those from major observational meta-analyses, confirming the generalizability of international evidence to Chinese populations and reinforcing the clinical value of PFO closure in rigorously selected patients. While several limitations related to study design and potential placebo effects should be acknowledged, the rapid onset of benefit with over 75% of responders improving within the first month, and the sustained efficacy during long-term follow-up further underscore its clinical utility. Beyond confirming efficacy, this study identifies two clinically meaningful predictors of procedural success.

First, a strong age-related trend in treatment response was observed. The responder rate was highest in the youngest cohort (15–24 years) at 89.67% and gradually declined with increasing age to 80.66% in the 45–54 years group. More notably, the quality of response differed markedly by age: the proportion of patients achieving Grade I (complete or near-complete) relief was 72.16% in the 15–24 years group but only 37.63% in the 55–65 years group. This age-dependent decline in both overall response and complete relief suggests that younger age is a clinically meaningful favorable factor, possibly due to greater neurovascular plasticity, less central sensitization from a shorter migraine history, and better-preserved cerebrovascular endothelial function. In multivariable logistic regression, age did not retain statistical significance as an independent predictor (OR = 0.95, *p* = 0.076). This attenuation may reflect interactions with other variables—for example, older patients in our cohort tended to have longer migraine durations and a higher burden of comorbidities such as hypertension or diabetes, which could partially mediate the age effect. Nevertheless, the consistent dose–response relationship across age groups and the clinically meaningful difference in complete relief rates support the continued use of age as an important patient selection criterion in clinical practice. This age-related efficacy trend is consistent with international studies and represents the first such quantification in a large Chinese PFO-migraine cohort.

Second, our analysis reveals that the chronological timing of the procedure is a significant independent predictor of outcome (OR = 0.89, *p* = 0.028), a novel finding not extensively reported previously. A critical methodological consideration in interpreting this finding is the cross-sectional nature of our follow-up design: patients underwent surgery at different time points between 2020 and 2024, and all were assessed at a single time point (study endpoint). Consequently, recent surgical cohorts (2023–2024) had shorter follow-up durations (e.g., 3–12 months) and were still on active DAPT, whereas earlier cohorts (2020–2022) had longer follow-up (up to 48 months) and had completed the DAPT regimen. The significantly higher responder rates observed in the most recent cohorts (2023–2024) likely reflect a combined effect of PFO closure and the antiplatelet and potential anti-inflammatory effects provided by the standardized postoperative DAPT regimen, particularly during the first 6–12 months. The anti-inflammatory and potential microemboli-reducing effects of DAPT may synergize with the closure mechanism, especially in the early postoperative period. This highlights a crucial point: the attributed benefit in non-randomized studies may be partially confounded by intensive concomitant medical therapy during the initial post-procedural phase. To directly dissociate the procedural effect from the pharmacological effect and evaluate the durability of mechanical closure alone, we examined the outcomes of patients who had completed DAPT.

Crucially, we examined the outcomes of earlier cohorts (2020–2022) who had completed DAPT and were followed for ≥12 months post-drug withdrawal. In these patients, the responder rate remained substantially high (ranging from 77.42% to 83.19% across Batch 4–8), with no evidence of a “rebound” phenomenon after medication cessation. This long-term sustained improvement after DAPT discontinuation strongly suggests that the therapeutic benefit of PFO closure is durable and not merely a transient drug effect in the Chinese population, addressing a key uncertainty for clinical practitioners in China. Furthermore, within these earlier cohorts, a significant inverse correlation was observed between procedure timing and responder rate: patients who underwent surgery earlier (e.g., 2020–2021) had modestly lower responder rates compared to those in late 2022–2023, even after accounting for the uniform DAPT background. While this finding likely reflects a learning curve effect with improved technical proficiency and perioperative management, it may also be influenced by more stringent patient selection over time. Nevertheless, this quantifiable association (OR = 0.89 per half-year delay, *p* = 0.028) underscores the importance of accumulated experience and systematized care as key drivers of long-term success. It critically highlights the importance of considering both pharmacological confounders and the learning curve when interpreting real-world outcomes of interventional procedures.

In contrast, our study found that other commonly considered variables, including patient sex, BMI, and occluder device type/model, did not significantly influence treatment response. This suggests that the mechanism of PFO-related migraine and its response to closure may be independent of these factors within our studied population. While the overall response rate is high, a subset of patients (≈16%) did not achieve significant relief. Our data preliminarily suggest that these non-responders may have distinct characteristics, such as older age and a BMI trending in the upper-normal to pre-obese range (23.8–25.1 kg/m^2^). More importantly, clinical observation from our cohort suggests that the presence of systemic comorbidities—such as hypertension, diabetes, or dyslipidemia—may be a key, yet often under-analyzed, factor associated with suboptimal outcomes in Chinese patients. The chronic endothelial dysfunction, pro-inflammatory state, and cerebrovascular structural changes associated with these conditions could impair the brain’s hemodynamic and neurovascular recovery after shunt elimination, thereby reducing the treatment effect. This observation, though requiring validation, points toward the future direction of precision medicine in this field: the development of predictive models to identify the minority of patients unlikely to benefit, thereby optimizing individual therapeutic decisions.

Beyond clinical and comorbidity-related factors, hypersensitivity to nitinol—the nickel–titanium alloy used in most PFO occluders—represents another plausible mechanism of non-response. Although we did not perform systematic allergy testing (e.g., patch testing or documented nickel allergy history) in this retrospective cohort, nickel hypersensitivity could theoretically trigger local inflammation, platelet activation, or endothelial injury, blunting the benefits of shunt closure. This mechanism has been postulated in atrial septal defect closure studies and may similarly apply to PFO closure. Future prospective protocols should incorporate preoperative nickel allergy screening to stratify risk and evaluate whether alternative (e.g., bioabsorbable or polymer-based) occluders improve outcomes in sensitized individuals.

Reduction of residual RLS after PFO closure is well established to correlate with reduced migraine burden, as complete shunt elimination is critical for sustained symptom relief. In complex PFO anatomy—particularly long-tunnel morphology (>10 mm) or those associated with atrial septal aneurysm (ASA)—achieving full closure remains challenging, with residual shunt rates up to 25% using standard techniques. Ilkay et al. (25) recently demonstrated that a detailed transseptal puncture (septostomy)-facilitated closure significantly mitigates this issue: in 144 patients with long-tunnel PFO, this technique yielded a very low residual shunt rate of only 1.4% at 6-month follow-up, alongside excellent procedural safety and sustained clinical improvement. In our cohort, all patients underwent standard PFO closure and achieved successful device implantation, with no clinically significant residual shunt detected at follow-up. Therefore, residual shunt is unlikely to be the primary driver of the observed 16% non-response rate in our study. While we did not employ septostomy-facilitated closure or conduct a dedicated quantitative analysis of residual shunt severity, we acknowledge this technique as a valuable adjunctive strategy to optimize closure efficacy and enhance migraine relief in patients with complex PFO anatomy. Nevertheless, given that even low-grade residual shunts have been linked to persistent migraine in some reports (26), future prospective studies should adopt standardized quantitative grading for residual shunt, correlate shunt severity with migraine outcomes, and further validate the clinical role of septostomy-facilitated closure in refractory cases.

The excellent safety profile observed—100% technical success, no major complications, and only 3.49% minor complications—strongly supports the feasibility of offering this intervention to a generally young and otherwise healthy migraine population. The minor complications were mechanistically understandable and manageable: access site issues related to peri-procedural antithrombotic management, transient arrhythmias from catheter/device mechanical stimulation, and self-limiting low-grade fever potentially linked to device-related inflammatory response. The absence of device-related thrombosis, stroke, or device embolization in patients with follow-up extending up to 48 months is reassuring for long-term management of Chinese PFO-migraine patients, a key consideration for routine clinical application.

## 5 Limitations of the study

Study limitations include its non-blinded, retrospective, single-center design and absence of a sham-control group, which introduce potential selection and assessment bias and limit the ability to fully account for strong placebo effects in migraine interventions. We also acknowledge that dual antiplatelet therapy (DAPT), especially aspirin, may have anti-inflammatory and analgesic properties that could theoretically contribute to migraine symptom relief. To mitigate these concerns, our cohort was restricted to patients with severe baseline disease burden (HIT-6 ≥ 60) and moderate-to-large RLS (cTCD grade ≥II), minimizing population heterogeneity. Efficacy was evaluated using objective composite endpoints (≥50% reduction in HIT-6 score or monthly migraine days), reducing subjective assessment bias. Critically, the responder rate remained 81.93% in patients followed >12 months after dual antiplatelet therapy discontinuation. Since placebo effects in migraine typically diminish within 3–6 months (27), this sustained benefit argues against a dominant placebo or antiplatelet effect. Thus, the observed high efficacy is unlikely to be primarily driven by DAPT. Moreover, the cross-sectional follow-up design, with a single assessment at variable postoperative time points, precluded observation of individual-level longitudinal changes. Furthermore, without precise follow-up dates, we used surgery date batches as a proxy, introducing minor approximation. Data on symptom onset were collected retrospectively and may be subject to recall bias. We also did not perform detailed subgroup analysis based on migraine subtypes (e.g., with *vs.* without aura), which is known to influence response. No objective biomarkers or advanced imaging were analyzed to clarify mechanisms of response. Additionally, unreported lifestyle and psychological factors were not assessed. Nevertheless, the large sample size and long-term follow-up (up to 48 months) provide robust real-world evidence for Chinese patients. While a sham-controlled trial is ideal, ethical and practical constraints limit its feasibility in this severely affected population. Future prospective, randomized studies in China with detailed phenotyping, adjuvant medication control, and multimodal biomarkers are warranted to refine patient selection and clarify the pure procedural effect.

## 6 Conclusion

Transcatheter PFO closure is a highly effective and safe treatment for Chinese patients with migraine and concomitant PFO. Maximum benefit is observed in younger individuals and in patients undergoing surgery in more recent years, as evidenced by the higher responder rates in later surgical batches. These findings strongly support the integration of this intervention into the therapeutic armamentarium for select Chinese patients with disabling, medically refractory migraine, and provide evidence-based guidance for patient selection in Chinese clinical practice. Future research should focus on precisely defining the optimal candidate and conducting subgroup analysis based on migraine subtypes to further improve treatment stratification.

## Data Availability

The raw data supporting the conclusions of this article will be made available by the authors, without undue reservation.
